# Broadband 75–85 MHz radiofrequency fields disrupt magnetic compass orientation in night-migratory songbirds consistent with a flavin-based radical pair magnetoreceptor

**DOI:** 10.1007/s00359-021-01537-8

**Published:** 2022-01-12

**Authors:** Bo Leberecht, Dmitry Kobylkov, Thiemo Karwinkel, Sara Döge, Lars Burnus, Siu Ying Wong, Shambhavi Apte, Katrin Haase, Isabelle Musielak, Raisa Chetverikova, Glen Dautaj, Marco Bassetto, Michael Winklhofer, P. J. Hore, Henrik Mouritsen

**Affiliations:** 1 Institute for Biology and Environmental Sciences, Carl von Ossietzky University Oldenburg, 26129 Oldenburg, Germany; 2 Centre for Mind/Brain Sciences, Università degli Studi di Trento, 38122 Trento, Italy; 3 Institute of Avian Research “Vogelwarte Helgoland”, 26386 Wilhelmshaven, Germany; 4 Institute for Physics, Carl von Ossietzky University Oldenburg, 26111 Oldenburg, Germany; 5 Department of Chemistry, University of Oxford, Oxford, OX1 3QZ UK; 6 Research Center Neurosensory Science, Carl von Ossietzky University of Oldenburg, Oldenburg, Germany

**Keywords:** Radical pair mechanism, Magnetoreception, Bird orientation, Electrosmog, Broadband electromagnetic fields

## Abstract

The light-dependent magnetic compass sense of night-migratory songbirds can be disrupted by weak radiofrequency fields. This finding supports a quantum mechanical, radical-pair-based mechanism of magnetoreception as observed for isolated cryptochrome 4, a protein found in birds’ retinas. The exact identity of the magnetically sensitive radicals in cryptochrome is uncertain in vivo, but their formation seems to require a bound flavin adenine dinucleotide chromophore and a chain of four tryptophan residues within the protein. Resulting from the hyperfine interactions of nuclear spins with the unpaired electrons, the sensitivity of the radicals to radiofrequency magnetic fields depends strongly on the number of magnetic nuclei (hydrogen and nitrogen atoms) they contain. Quantum-chemical calculations suggested that electromagnetic noise in the frequency range 75–85 MHz could give information about the identity of the radicals involved. Here, we show that broadband 75–85 MHz radiofrequency fields prevent a night-migratory songbird from using its magnetic compass in behavioural experiments. These results indicate that at least one of the components of the radical pair involved in the sensory process of avian magnetoreception must contain a substantial number of strong hyperfine interactions as would be the case if a flavin–tryptophan radical pair were the magnetic sensor.

**Supplementary Information:**

The online version contains supplementary material available at 10.1007/s00359-021-01537-8.

## Introduction

The magnetic compass of night-migratory songbirds (Wiltschko 1968) is an inclination compass (Wiltschko and Wiltschko 1972) that is light dependent (Wiltschko et al. 1993; Zapka et al. 2009; Mouritsen 2018) and involves the birds’ visual system (Mouritsen et al. 2005, 2016; Heyers et al. 2007; Zapka et al. 2009, 2010). Most evidence suggests that the sensory mechanism is based on a radical pair process (Schulten et al. 1978; Ritz et al. 2000; Hore and Mouritsen 2016; Xu et al. 2021), but the primary magnetic sensors have yet to be unequivocally identified. The birds’ ability to orient using their magnetic compass is dependent on the wavelength of the ambient light (Wiltschko et al. 1993, 2010; Mouritsen 2018; but see Kirschvink et al. 2010). Furthermore, very weak broadband radiofrequency (RF) fields from  ~ 100 kHz to  ~ 10 MHz prevent the birds from using their magnetic compass (Ritz et al. 2004; Engels et al. 2014; Schwarze et al. 2016; Kobylkov et al. 2019). Disturbing effects of single-frequency RF fields have also been reported (Thalau et al. 2005; Kavokin et al. 2014; Pakhomov et al. 2017; Bojarinova et al. 2020), although Schwarze et al. (2016) found that broadband fields, even at substantially lower intensities, had a much stronger effect. Any putative magnetite-based sensor should not be affected by RF fields of the low intensity and frequency range used in the behavioural experiments (Kirschvink 1996; Ritz et al. 2000). In contrast, a radical-pair-based mechanism seems to be consistent with a light-dependent magnetic sensor susceptible to weak RF fields at low MHz frequencies (Solovyov et al. 2014; Hore and Mouritsen 2016).

Cryptochromes (Cry), currently the only group of vertebrate proteins known to form radical pairs upon photoexcitation (Ritz et al. 2000; Liedvogel et al. 2007; Maeda et al. 2012; Zoltowski et al. 2019; Xu et al. 2021), have been proposed as the light-activated, radical-pair-forming, magnetosensory proteins in the avian retina (Ritz et al. 2000; Mouritsen et al. 2004; Maeda et al. 2012; Nießner et al. 2014; but see Bolte et al. 2021; Hore and Mouritsen 2016; Günther et al. 2018; Xu et al. 2021; Wong et al. 2021). At least six different cryptochrome variants are known to occur in retinal neurons of night-migratory songbirds: Cry1a (Mouritsen et al. 2004; Möller et al. 2004; Nießner et al. 2011; Bolte et al. 2021), Cry1b (Möller et al. 2004; Bolte et al. 2016; Nießner et al. 2016), Cry2a (Mouritsen et al. 2004; Möller et al. 2004; Balay et al. 2021; Einwich et al. 2021), Cry2b (Hochstoeger et al. 2020; Balay et al. 2021), Cry4a (Liedvogel and Mouritsen 2010; Günther et al. 2018; Wu et al. 2020; Xu et al. 2021), and Cry4b (Einwich et al. 2020); a and b forms are alternative splicing variants.

Avian versions of Cry1 and Cry2 seem to be unable to bind flavin adenine dinucleotide (FAD) (Kutta et al. 2017; Wang et al. 2018; Hochstoeger et al. 2020). Without FAD, cryptochromes do not absorb visible light, cannot form intramolecular radical pairs, and consequently are not magnetically sensitive. In contrast, avian Cry4 variants bind FAD stoichiometrically (Ozturk et al. 2009; Mitsui et al. 2015; Wang et al. 2018; Zoltowski et al. 2019; Hochstoeger et al. 2020; Xu et al. 2021). Even more importantly, Cry4a from night-migratory European robins (*Erithacus rubecula*) was recently shown to be magnetically sensitive in vitro (Xu et al. 2021). Following photoexcitation of the bound FAD, the magnetically sensitive radical pair forms in vitro by the sequential hopping of an electron along a chain of four tryptophans (Trp) (Giovani et al. 2003; Müller et al. 2015; Nohr et al. 2016, 2017; Zoltowski et al. 2019; Xu et al. 2021). This radical pair in European robin Cry4a was shown to be [FAD^·−^ TrpH^·+^] (Xu et al. 2021). Both of these radicals have more than ten magnetic nuclei (^1^H and ^14^N) hyperfine-coupled to their unpaired electrons (Hore and Mouritsen 2016).

Other studies suggest that a FAD^·−^-Z^·^ or FAD^·−^-X^·^ radical pair, in which the second part of the radical pair has no (Z^·^) or very few (X^·^) hyperfine interactions, could be responsible for sensing magnetic compass information in night-migratory songbirds. Indirect evidence for this hypothesis includes (a) reports of specific RF field effects at the electron Larmor frequency (Ritz et al. 2004, 2009; Thalau et al. 2005; Kavokin et al. 2014; Bojarinova et al. 2020; but see Engels et al. 2014; Schwarze et al. 2016); (b) indications that a “dark radical reaction”, perhaps involving superoxide, could be responsible for magnetoreception (Müller and Ahmad 2011; Wiltschko et al. 2016; Pooam et al. 2019; but see Player and Hore 2019); and (c) results of behavioural experiments in which birds were subjected to alternating pulses of light and magnetic field (Wiltschko et al. 2016). An attractive feature of this hypothesis is that a FAD^·−^-Z^·^ or FAD^·−^-X^·^ radical pair could be more sensitive to Earth-strength magnetic fields than a FAD^·−^-TrpH^·+^ radical pair (Lee et al. 2014). However, all of these findings are controversial, and, in many cases, we have been involved in replication studies which led to contradictory results. For instance, in our previous experiments (Schwarze et al. 2016), exposure to RF fields at the Larmor frequency did not elicit disruptive effects on the magnetic orientation of birds. Instead, we found that broadband RF fields are more effective at disrupting orientation performance compared to single-frequency RF fields, which conflicts with predictions for FAD^·−^-Z^·^ (Ritz et al. 2004; Engels et al. 2014; Schwarze et al. 2016). Another problem is that the identity of the Z^·^ or X^·^ radical is completely unknown (Lee et al. 2014). Superoxide, which would have no hyperfine interactions, has been suggested, but seems highly unlikely because of its extremely fast spin relaxation (Hogben et al. 2009; Player and Hore 2019; but see Kattnig 2017) and ascorbic acid does not appear to have the appropriate binding properties to lead to a viable radical pair (Nielsen et al. 2017).

**Fig. 1:**
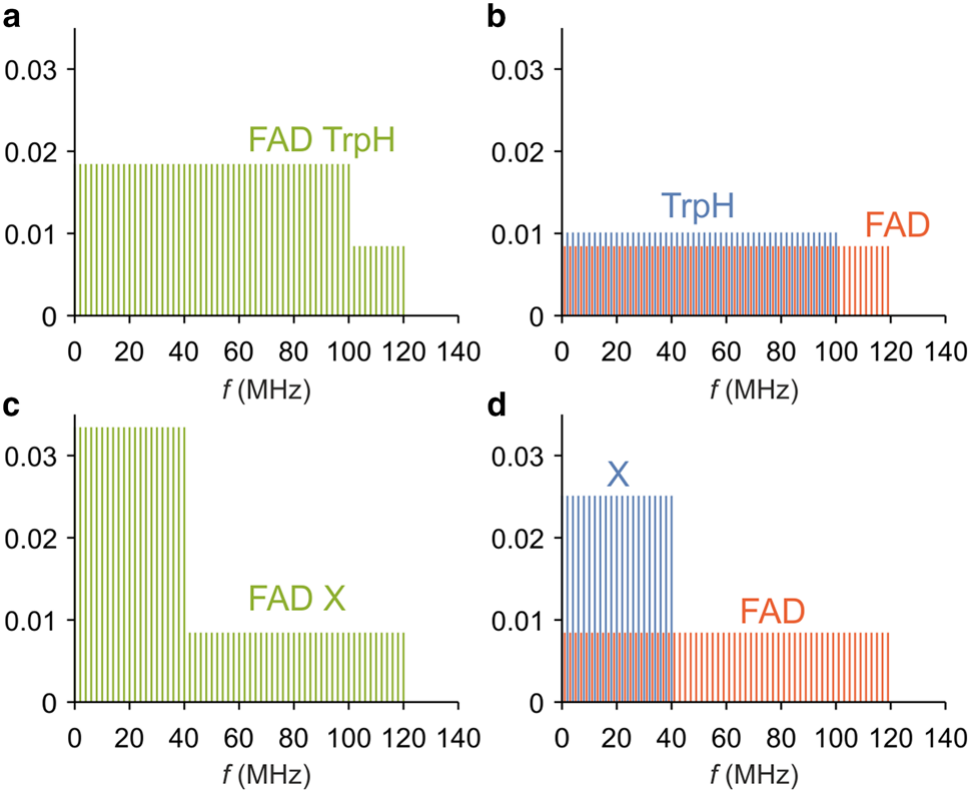
Simplified simulated action spectrum histograms of FAD-containing radical pairs showing the expected resonances for radical pairs (**a**, **c**) and their constituent radicals (**b**, **d**). FAD^·−^-TrpH^·+^ (**a**, **b**). FAD^·−^-X^·^ (**c**, **d**). The spectra in b and d have been separately normalized. **a** and **c** are the sums of the red and blue spectra in **b** and **d**, respectively

An impression of the likely effects of RF magnetic fields on different radical pairs can be gleaned from the “action spectrum histograms” introduced by Hiscock et al. (2017). Calculated from the hyperfine interactions within the radicals and the dipolar coupling between them, the plots in Hiscock et al. (2017; Figs. 3a,  4a,  5a,  6a) show the approximate relative sensitivity of a radical pair to applied RF fields of different frequencies. Each histogram in Hiscock et al. (2017) and ours in Fig. 1 are normalised such that the heights of the bars sum to unity. The taller the bar, the larger the expected effect at that frequency. The frequencies at which a radical pair can be influenced by a time-dependent field are given by the gaps between pairs of the quantized energy levels of its coupled electron-nuclear spin system. An important point to come from these plots is that each radical has a “cut-off” frequency above which it can no longer be in resonance with, and therefore affected by, a RF field. These upper limits were estimated to be  ~ 120 MHz for FAD^·−^ and  ~ 100 MHz for TrpH^·+^ (Hiscock et al. 2017). Ignoring the electron exchange and dipolar couplings, the cut-off frequency for a radical pair is the larger of the cut-offs of the two radicals. In the case of FAD^·−^-TrpH^·+^ this would be  ~ 120 MHz.

This concept is illustrated in Fig. 1a which shows a simplified simulated version of the histogram for a FAD^·−^-TrpH^·+^ radical pair, ignoring the electron–electron spin–spin interactions. As the radiofrequency is increased from  ~ 1 MHz, the predicted response is fairly flat up to the maximum frequency for TrpH^·+^ (100 MHz), then drops by about 50%, before falling to zero at 120 MHz. The origin of this behaviour can be seen in Fig. 1b which shows the contributions from the two radicals. The normalised histogram bars for TrpH^·+^ are about 20% higher than those of FAD^·−^ because of the lower cut-off frequency. The reduced response between 100 and 120 MHz in Fig. 1a arises because both radicals can be in resonance with RF fields at frequencies below 100 MHz, but only FAD^·−^ is affected in the range 100–120 MHz. The effect of a small dipolar interaction is to smooth the spectrum in Fig. 1a, making the drop at 100 MHz less abrupt and therefore probably very difficult to detect experimentally. One can, therefore, expect FAD^·−^-TrpH^·+^ to show broadly the same sensitivity to any frequency in the range 1–120 MHz and to be almost unaffected by frequencies above 120 MHz.

Figure 1c, d shows the corresponding plots for a FAD^·−^-X^·^ radical pair in which X^·^ has fewer hyperfine interactions and therefore a lower cut-off frequency than TrpH^·+^ (chosen here, arbitrarily, to be 40 MHz). The step change at 40 MHz in Fig. 1c is much more pronounced than that at 100 MHz in Fig. 1a because the contributions of the two radicals (Fig. 1d) are now much more distinct. In this case, one could expect that RF fields in the range 1–40 MHz should have a larger effect than those in the range 40–120 MHz. Once again, there would be no effect at frequencies beyond 120 MHz. This prediction is unlikely to be changed much when a small dipolar interaction is introduced. Both FAD^·−^-TrpH^·+^ and FAD^·−^-X^·^ are predicted to be sensitive to 75–85 MHz fields.

The aim of the present study is to test whether a broadband RF field in the 75–85 MHz range can disturb the magnetic compass orientation of Eurasian blackcaps (*Sylvia atricapilla*), a night-migratory songbird, and thus whether at least one of the radicals involved in the bird’s magnetic compass sense contains more than about 10 hyperfine interactions. Failure to observe a disorienting effect of 75–85 MHz fields at RF field strengths similar to those that cause disorientation at lower frequencies would be evidence against a flavin-containing radical pair sensor.

## Methods

The experiments were conducted at the University of Oldenburg, Germany, during the spring migratory seasons of 2019 and 2021 using two different cohorts of Eurasian blackcaps which were wild-caught after the breeding season and during autumn migration in the immediate vicinity of the University. The birds were kept in on-site cages in a windowless room under a light regime imitating the local photoperiod and had access to food and water ad libitum. Between migratory seasons, the birds were kept in an indoor aviary with an outdoor option.

### Testing site

Behavioural experiments took place in specially constructed non-magnetic laboratory buildings described in earlier studies (Kobylkov et al. 2019; full description in Schwarze et al. 2016). Within the laboratory, three aluminium-shielded chambers acting as Faraday cages allowed static magnetic fields to pass through, while attenuating time-dependent electromagnetic fields, ranging from 10 kHz to 10 GHz, by a factor of at least 10^5^. The electrical equipment used to generate the RF fields was grounded through an 8 m-deep earthing rod, while the individual chambers were grounded by use of single electrode loops in the laboratory base (Schwarze et al. 2016).

### Generation and measurement of static magnetic field stimuli

Behavioural experiments were performed in two static magnetic field conditions, the normal magnetic field (NMF) and a changed magnetic field (CMF), in which the horizontal component of the field was rotated 120° counter-clockwise. Static magnetic fields were generated by a double-wrapped, three-axis Merritt four-coil system in each of the three chambers (Kirschvink 1992; Mouritsen 2013; Schwarze et al. 2016). The rectangular coils measured ca. 2 × 2 m. The behavioural experiments were conducted on a wooden table in the centre of the coil system where the homogeneity of the magnetic field was at least 99%. Each of the three sets of four coils was powered by a separate constant-current power supply (BOP 50–4 M, Kepco Inc., Flushing, NY, USA). In each chamber, the local and 120°-counter-clockwise rotated magnetic fields were recorded daily at alternating, opposite corners and in the centre of the experimental table using a flux-gate magnetometer (FVM-400, Meda Inc., Dulles, VA, USA).

The NMF condition was generated by running anti-parallel currents through the double-wrapped coils. Because the magnetic fields induced by currents running in opposite directions through the two halves of the windings in each coil exactly cancel one another, the birds perceived only the natural local geomagnetic field. In the CMF condition, the currents ran parallel through the double-wrapped coils, reinforcing instead of cancelling the fields they generated. The average magnetic measurements of the NMF over all test days and chambers were inclination 67.58° ± 0.35° (mean  ±  sd) and intensity 48,827.04 ± 344.97 nT. The average CMF measurements were inclination 67.59° ± 0.33°, horizontal direction (declination)—119.97° ± 0.78° and intensity 48,808.83 ± 327.01 nT.

### Generation and measurement of time-dependent electromagnetic fields

**Fig. 2:**
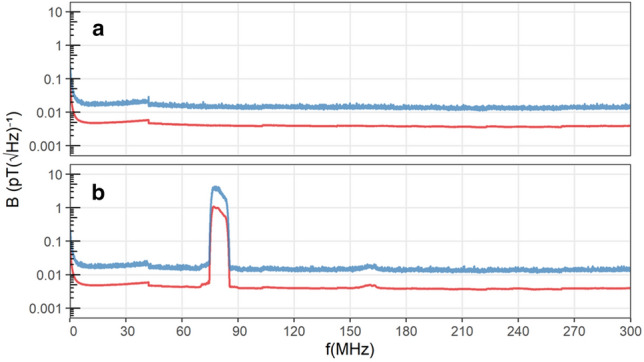
Measurements of the magnetic components of the RF fields measured in the range of 150 kHz–300 MHz. **a** The control condition and **b** the 75–85 MHz broadband noise used for the “RF” condition. Electric component spectra are displayed in Fig. SI1 in the Supplementary Information. Spectral traces: ‘average’ (lower red line); ‘maxhold’ (upper blue line). Notice that the magnetic spectrum is extremely clean with no significant harmonics or other frequencies outside the desired range

Every day, after the daily measurement of the static magnetic field, the magnetic components of the RF fields were measured in each chamber at opposite corners or edges of the tables. As recommended by Hiscock et al. (2017), the fields were measured with a calibrated active loop antenna (Schwarzbeck Mess-Elektronik, HFS 1546, 150 kHz–400 MHz, Germany), placed 1.5 cm above the centre of the emission antennas. The measurement antenna was connected through the wall panel to a signal analyser (Rhode and Schwarz, FSV3004 Signal and Spectrum Analyser (10 Hz–4 GHz), Germany). The electric components of the fields were measured similarly with a calibrated active bi-conical antenna (Schwarzbeck Mess-Elektronik, EFS 9218 (9 kHz–300 MHz), Germany), connected to the signal analyser. The RF fields were recorded for 1 min daily with the resolution bandwidth of the analyser set to 10 kHz. Before and after a migratory season, a 1-h measurement took place to record the RF fields applied during the experimental phase (magnetic component: Fig. 2; electric component: Fig. SI1). For comparability with earlier studies (Engels et al. 2014; Schwarze et al. 2016; Kobylkov et al. 2019), we recorded two different spectral traces in each measurement: maxhold (with the “Maxpeak” detector; for the detection of absolute maxima at a given frequency) and average spectral amplitudes (with the “RMS” detector; measuring the root-mean-square intensity over time at a given frequency). The spectral density (pT/√Hz) of the applied broadband noise signal in the frequency range of interest was integrated according to the equations in Kobylkov et al. (2019), see Table SI1.

The RF fields were generated with signal generators (Rhode and Schwarz, SMBV100A (9 kHz–6 GHz) and SMBV100B (8 kHz–3 GHz), Germany), set to produce broadband noise in the spectra range from 75 to 85 MHz (centre frequency: 80 ± 5.75 MHz, resulting in a signal plateau width of 10 MHz). In the treatment condition (in the following noted as “RF”), the output RMS voltage at the signal generator was set to 37 mV, while in the control condition, the RMS voltage was set to 15 nV, so that in both conditions, the signal generators were actively producing a signal. Unless noted otherwise, coaxial cables (Schwarzbeck Mess-Elektronik, AK 9515 E, 50 Ohm, N-Connector, Germany) with N-connectors were used.

The signals were then fed into broadband power amplifiers (AR Deutschland GmbH, 30W1000-M7 (20 MHz–1 GHz) and 50U1000 (10 kHz–1000 MHz), Germany) and amplified to 55% of the maximum gain (ca. 45 dB) and guided into the experimental chambers via a wall panel. Inside the chambers, the signal was passed to a custom-built bandpass filter box (attenuation up to 71.88 MHz; 3 dB attenuation at 71.88 MHz), followed by an 8-Way splitter (Werlatone, Model D5829-10, 20–500 MHz, 400 W, N-Connectors, Patterson, NY, USA). Both were located under the experimental table within the Merritt-4 coil system. Each of the eight outputs from the splitter was fed into a coaxial cable (RG58C/U MIL-C-17, BNC connector), which was wrapped in a single turn around a custom-built, circular PVC antenna frame (diameter: 35.7 cm, height: 9 cm, circumference: 112.2 cm). The shield of the coaxial cable was removed along the circumference of the single turn coil, and the inner conductor was connected at its end to the shield at the other end to close the loop. This single-loop magnetic coil acted as an antenna, applying the generated broadband noise to the Emlen-funnel placed inside. The generators were in ‘RF OFF’-mode and only switched to ‘RF ON’-mode for the experiments or during measurements.

### Acquisition and analysis of behavioural data

**Fig. 3:**
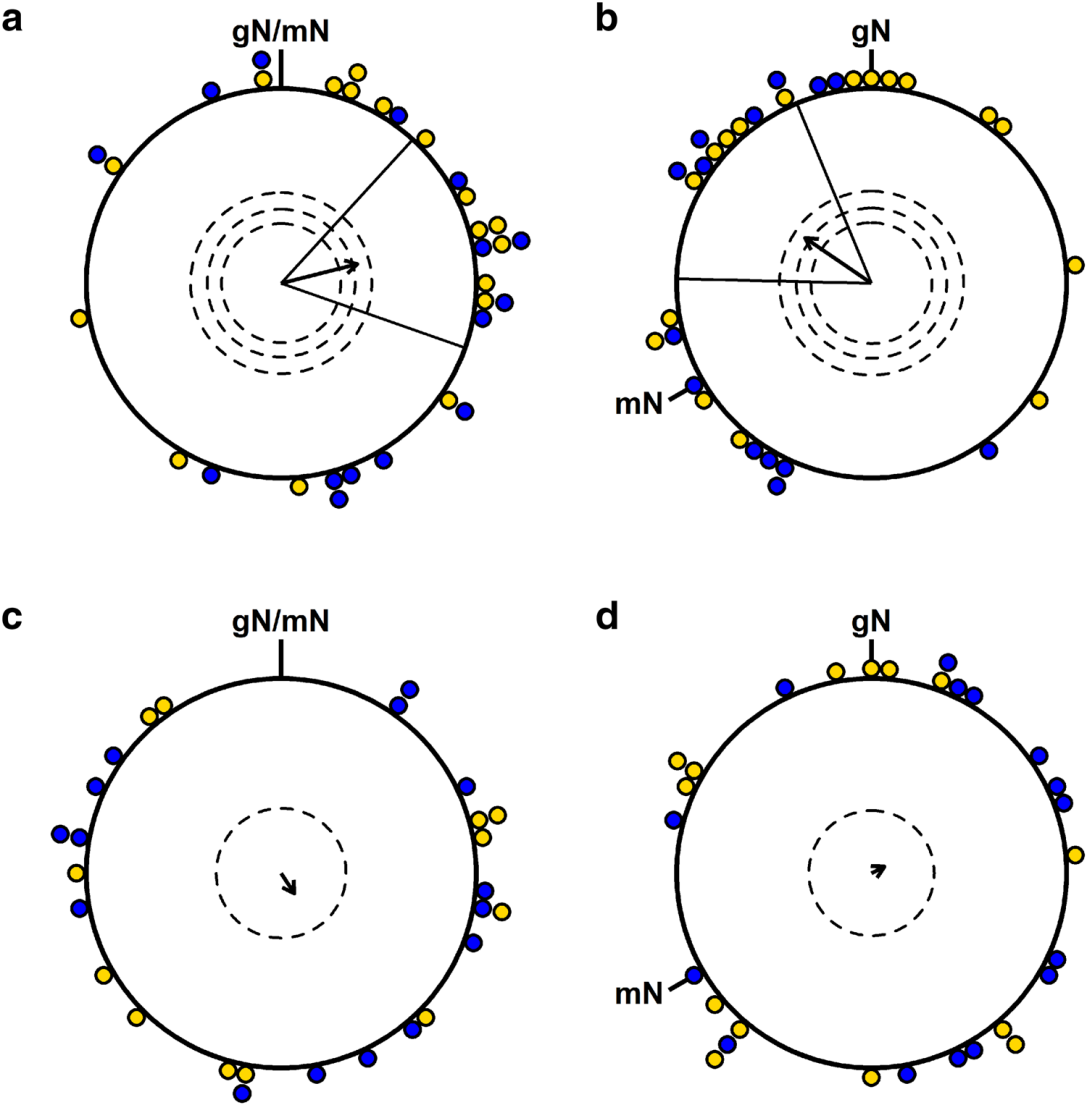
Magnetic compass orientation of Eurasian blackcaps. **a** NMF—normal Earth’s magnetic field in Oldenburg (*N*  = 32); **b** CMF—120°-rotated Earth’s magnetic field (*N*  = 31); **c** NMF-RF—NMF with 75 to 85 MHz radiofrequency (RF) fields present (*N*  = 27); **d** CMF-RF—CMF with 75–85 MHz RF fields present (*N*  = 29). Each coloured dot represents the mean direction of one individual bird rounded to the nearest 5°. The arrows display the group mean orientation and the arrow length represents the respective Rayleigh value, framed by the confidence intervals (± 95%). Dashed circles indicate threshold *p* levels (from inner to outer circle: 0.05, 0.01, 0.001) of the Rayleigh test for the corresponding sample size. *gN* geographical North; *mN* magnetic North. Yellow dots: birds from the 2019 cohort; blue dots: birds from the 2021 cohort. Each dot in a given condition represents data from different individuals. The same individuals were tested in all 4 conditions, but, in some cases, a bird did not provide enough active and directed tests in all conditions. Therefore, the sample size is slightly different between the conditions

Eurasian blackcaps were pre-screened by testing each individual’s migratory motivation and ability to orient using their magnetic compass in the NMF and CMF conditions without RF-noise. The pre-screening results are not shown in Fig. 3, as these data were used for selection, and, thus, NMF and CMF data without RF-noise needed to be collected again for the test series reported in Fig. 3. Therefore, having completed the pre-screening tests, the actual experiments were conducted from scratch. On every test day, the birds were caught from the housing cages 1 h before the end of civil twilight (approximately 30 min before sunset) to allow them to experience the sunset and potentially to calibrate their magnetic compass (Cochran et al. 2004, but see Chernetsov et al. 2011). The birds were placed in the nearby botanical garden facing the sunset and sheltered from rain and wind. The boxes in which they were transferred to the nonmagnetic experimental laboratory were covered to exclude any disturbance from street lights.

Behavioural experiments were performed using modified Emlen funnels made of white PVC (35 cm diameter, 15 cm high, walls 45° inclined) (Emlen and Emlen 1966; Mouritsen et al. 2009). During the migratory season, night-migratory songbirds exhibit “migratory restlessness”, expressed as jumping around and wing-whirring in a cage. During periods of migratory restlessness at night, the birds jump in the direction they want to migrate. The sloping sides of the funnels cause them to slide back down leaving marks on the scratch-sensitive thermal paper with which the funnels are lined. In this way, the birds’ intended migratory directions could be recorded. The edges of the scratch papers were joined with adhesive tape and the resulting overlaps aligned with one of the cardinal directions, chosen at random for each experiment and measurement chamber.

In the experimental chambers, each bird was placed in a designated Emlen funnel lined with scratch-sensitive thermal paper. After 1 h, the birds were returned to their transport boxes. Every test day consisted of two rounds of the same experimental condition with only the position of the funnel on the experimental table changed between rounds for each bird. After the second experimental round, the birds were brought back to their housing cages. After every experimental round, the scratch papers were collected, the funnels cleaned and, if necessary, prepared for another round.

The scratch papers were analysed independently by two researchers, unaware of the static magnetic field and RF field conditions, as well as the cardinal alignment of the scratch papers. Papers that had fewer than 30 scratches (203 of 939 papers, 21.6%) were classified inactive and excluded from further analysis. For active birds, the mean orientation of their scratches was determined to the nearest 10°. If the orientations estimated independently by the two researchers agreed to within 30°, the mean of the two values was taken as the bird’s orientation (626 of 939 papers, 66.7%). Otherwise, the paper was reassessed and if the two directions still differed by more than 30°, the paper was deemed to be random (110 of 939 papers, 11.7%). For every bird in each condition, its mean direction and directedness (*r*: mean resultant vector length) of all individual test results were calculated with a custom-written R-script (R Core Team 2013; “circular” package: Agostinelli and Lund 2017; “tidyverse” package Wickham et al. 2019) using circular statistics. The data used for the final analysis are accessible in Table SI2 in the Supplementary Information.

For each bird, the mean orientation of all tests in a given treatment condition was calculated. In line with previous studies (Lefeldt et al. 2015; Kobylkov et al. 2019), we included the means of all individuals with at least 3 directed tests in the relevant condition and *r*  ≥ 0.2 in Fig. 3. We also required that any given individual bird provided a directional value in at least two of the four test conditions. For every experimental condition, the group’s mean orientation and directedness was then calculated by adding up unit vectors in each of the individual birds’ mean directions and dividing by the number of birds providing data for the given experimental condition. The group mean orientation was compared against uniformity with the Rayleigh test. The randomness of experimental groups that showed no statistically significant orientation was compared with directed experimental groups using bootstrapping (Fisher 1995; Alert et al. 2015; Bojarinova et al. 2020). For the bootstrap, we merged the data from both years and resampled the data a total of 100,000 times per condition randomly with replacement according to sample sizes of the respective controls in Fig. 3.

## Results

In the normal geomagnetic field of Oldenburg (NMF), the group orientation of the birds that had not been exposed to 75–85 MHz RF fields resembled the north-easterly spring migratory direction seen in free-flying conspecifics in the wild [NMF, Fig. 3a; *N*  = 32, mean  = 75.90°, standard deviation (sd)  = 77.37°; *r*  = 0.4018, *p*  = 0.0050; 95% confidence interval (CI)  ± 33.44°]. When the horizontal component of the magnetic field was turned by 120° counter-clockwise, the birds adjusted their orientation accordingly (CMF, Fig. 3b; *N*  = 31, mean  = 304.35°, sd  = 76.28°; *r*  = 0.4122, *p*  = 0.0044; 95% CI  ± 33.04°). The orientation in the rotated CMF field was significantly different from the orientation under NMF conditions [Mardia–Watson–Wheeler test: *ω*  = 17.419, degrees of freedom (*df*)  = 2, *p* = 0.0002], the two confidence intervals include the expected 120° counter-clockwise rotation, and the CMF directions were significantly distributed around the mean angle rotated counter-clockwise by 120° relative to the NMF control mean direction (V-Test against 315.9°: *V*  = 0.404, *µ*  = 3.18, *p*  = 0.0006).

Birds that were exposed to broadband 75–85 MHz RF fields at an intensity of 2.53pT/√Hz (maxhold mode) were randomly oriented as a group in both magnetic field conditions (NMF-RF, Fig. 3c; *N*  = 27, mean = 146.71°, sd  = 117.13°; *r*  = 0.1237, *p*  = 0.6655; CMF-RF, Fig. 3d; *N*  = 29, mean  = 62.88°, sd  = 130.73°; *r*  = 0.0740, *p*  = 0.8552).

To validate whether the orientation results obtained using 75–85 MHz RF noise (Fig. 3c, d) were significantly more random than in the two conditions in which no such fields were applied (Fig. 3a, b), a common bootstrap approach was applied (100,000 iterations per condition; Bojarinova et al. 2020; Fisher 1995). The bootstraps for the NMF-RF showed that only 0.854% (*p*  = 0.00854) of the bootstrap iterations achieved a directedness (*r*  ≥ 0.4018) as high as or higher than the NMF condition (its control counterpart). Only 0.41% (*p*  = 0.0041) also lay within the confidence intervals (42.46°–109.34°) of the NMF. The CMF-RF condition bootstrap data yielded similar results relative to the respective parameters of the CMF condition, with only 0.902% (*p * = 0.00902) being as directed as the CMF data (*r*  ≥ 0.4122) and only 0.019% (*p*  = 0.00019) also within the confidence intervals. Since all bootstrap results were below 1%, the orientation of the birds exposed to the 75–85 MHz noise were significantly more random than the birds in the control conditions.

## Discussion

When tested under control conditions, the birds exhibited magnetic compass orientation: they displayed a seasonally appropriate group orientation in the normal magnetic field (NMF) condition and they adjusted their group heading according to the horizontally rotated magnetic field, CMF. When 75–85 MHz RF fields were applied, the mean orientation of the blackcaps as a group was indistinguishable from random in both the NMF and CMF conditions.

**Fig. 4:**
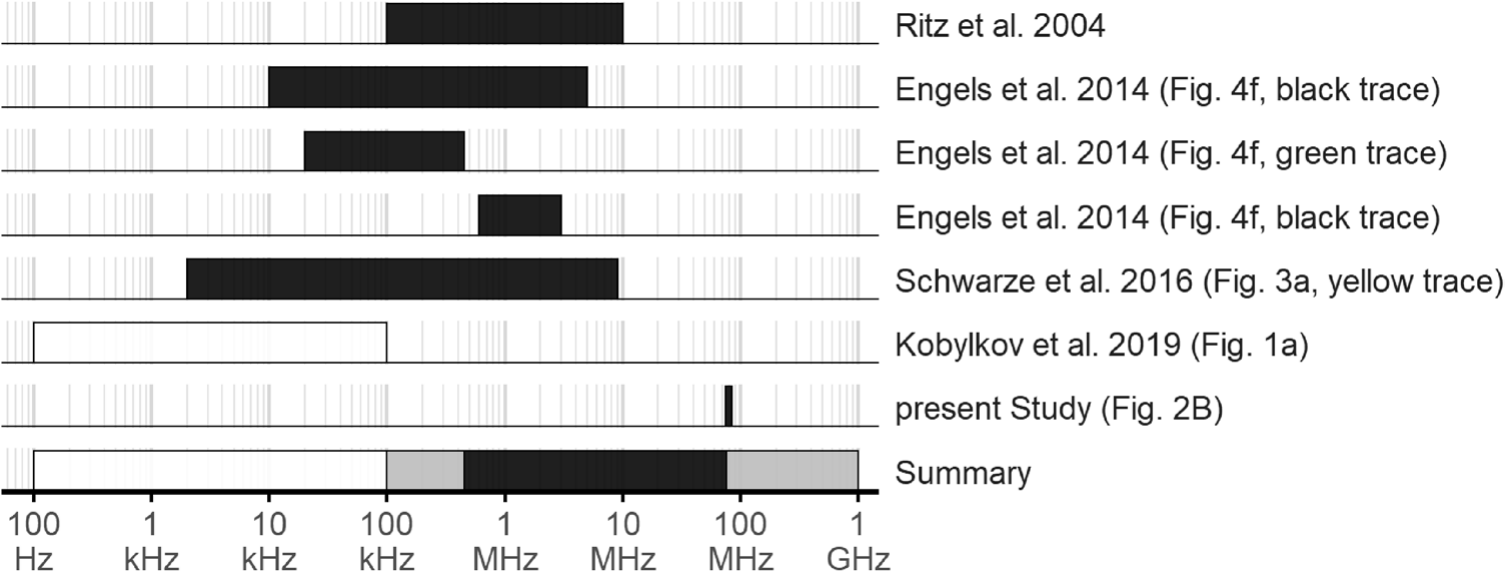
Summary of the effects of broadband RF fields on the orientation behaviour of night-migratory songbirds. Black boxes indicate that a disruptive effect was reported, white boxes indicate no disruptive effect. The grey boxes in the summary row indicate the uncertainty in the lowest and highest frequencies known to cause disorientation. Note that the horizontal axis is logarithmic and that the data from the present study spans 10 MHz, similar to the previous studies. Based on these data, RF fields at least in the range from about 400 kHz to about 80 MHz seems to disrupt magnetic compass orientation in the night-migratory songbird species tested so far

Our results are in line with previous research (Ritz et al. 2004; Engels et al. 2014; Schwarze et al. 2016) showing that a broadband RF noise can disturb magnetic compass orientation of night-migratory songbirds (summarised in Fig. 4). The frequency range of the broadband fields that have this disruptive effect starts somewhere between 100 kHz (Kobylkov et al. 2019) and 450 kHz (Engels et al. 2014) and, given the results presented here, ends above 75 MHz.

Disorientation at 75–85 MHz is consistent with a flavin-containing, radical-pair-based, magnetosensory mechanism. It will be interesting to further investigate whether the high-frequency cut-off, above which the birds are no longer sensitive to RF fields, is indeed in the region of 120 MHz. This range would be expected for a flavin-containing radical pair (see Fig. 1). If the upper limit of the RF effect were shown to occur at a substantially higher frequency (e.g., > 200 MHz), this would imply that one or both of the radicals would need to have much larger hyperfine interactions than either FAD^·−^ or TrpH^·+^ or that the sensory mechanism is based on an unknown mechanism sensitive to high-frequency RF noise.

The results reported here cannot distinguish between FAD^·−^-TrpH^·+^ and FAD^·−^-Z^·^ or FAD^·−^-X^·^ radical pairs as the source of the RF effect. To discriminate between these possibilities, one would need to detect either the drop in sensitivity at  ~ 100 MHz when the cut-off for TrpH^·+^ is exceeded (Fig. 1a) or the same effect at a lower frequency above which X^·^ is no longer affected (shown, arbitrarily, at 40 MHz in Fig. 1c). These relatively small changes in magnetic response sensitivity are on the order of a factor 2 or 3 and are expected to be gradual. Therefore, any experiments would first require the identification of the exact RF cut-off amplitude at around 10 MHz, which is currently only known within about two orders of magnitude (see Supplementary Information, Table SI1). In our view, due to the directional noise and variability in behavioural experiments, it is unlikely that behavioural experiments can provide the sensitivity to identify these relatively subtle changes in magnetic responses. If widely reproducible electrophysiological responses from magnetically sensitive neurons in night-migratory songbirds could be recorded, such an approach would be much more sensitive and provide much faster and verifiable results regarding these matters than behavioural experiments could.

## Supplementary Information

Below is the link to the electronic supplementary material.

Supplementary file 1 (PDF 1578 KB)

## Data Availability

Original data are available in the electronic supplementary material.
