# Supplementary material for: Broadband 75–85 MHz radiofrequency fields disrupt magnetic compass orientation in night-migratory songbirds consistent with a flavin-based radical pair magnetoreceptor
Source: J Comp Physiol A Neuroethol Sens Neural Behav Physiol. 2022 Jan 12;208(1):97–106. doi: 10.1007/s00359-021-01537-8 (PMC8918455; doi:10.1007/s00359-021-01537-8)
Supplement: Supplementary file 1 — (PDF 1578 KB) [file 359_2021_1537_MOESM1_ESM.pdf]

Title:

Broadband 75-85 MHz radiofrequency fields disrupt magnetic compass orientation in night-migratory songbirds consistent with a flavin-based radical pair magnetoreceptor

Journal:

Journal of Comparative Physioloy A

Authors:

Bo Leberecht, Dmitry Kobylkov, Thiemo Karwinkel, Sara Döge, Lars Burnus, Siu Ying Wong, Shambhavi Apte, Katrin Haase, Isabelle Musielak, Raisa Chetverikova, Glen Dautaj, Marco Bassetto, Michael Winklhofer, P. J. Hore and Henrik Mouritsen

Corresponding author:

Henrik Mouritsen - Henrik.Mouritsen@uni-oldenburg.de

Institute for Biology and Environmental Sciences, Carl von Ossietzky University Oldenburg, Oldenburg, 26129, Germany.

Research Center Neurosensory Science, Carl von Ossietzky University of Oldenburg, Oldenburg, Germany.

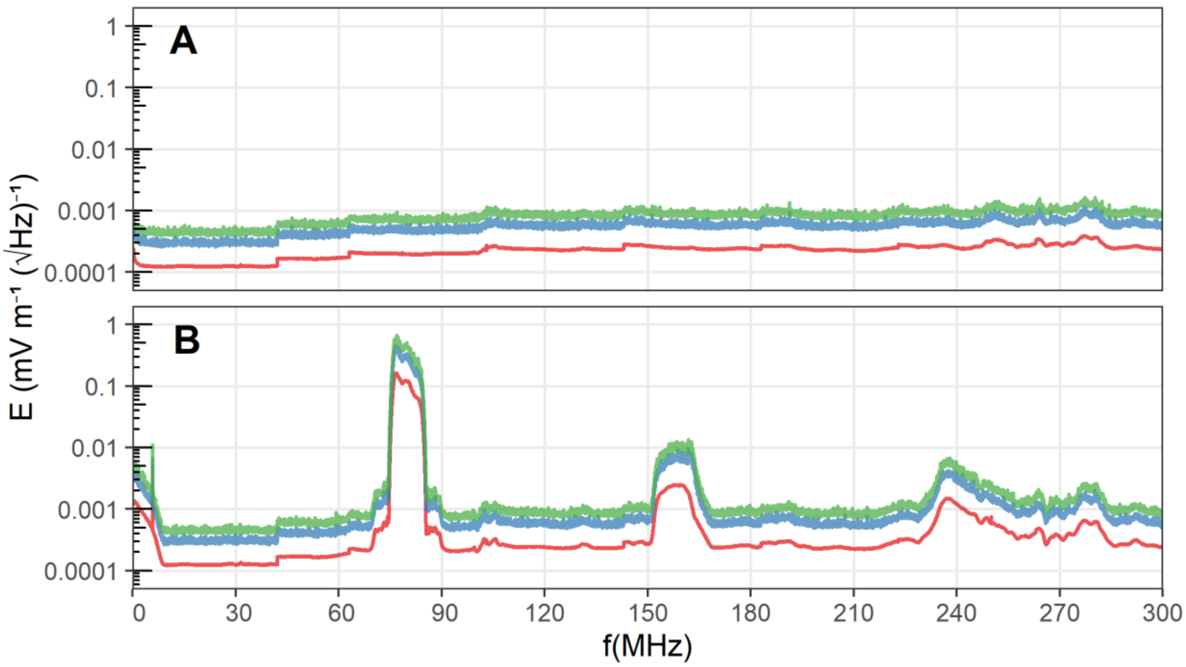

Fig. SI1: Measurements of the electric components of the RF fields measured in the range of 150 kHz to 300 MHz. (A) The control condition and (B) the 75-85 MHz broadband noise used for the “RF” condition. Magnetic component spectra are displayed in Fig. 2 in the main text. Spectral traces: ‘average’ (lower red line); ‘maxhold’ (upper blue line). Notice that the electric disturbance spectrum shows harmonic impurities, in contrast to the extremely clean magnetic components in Fig. 2.

Table SI1: Comparative table of different density and intensity measures from (Kobylkov et al. 2019), with measures of present study appended. Species: ER: European robin, BC: Eurasien blackcap. For formulas of $\bar{b}^{\max}$, $\bar{b}$, $B_{\mathrm{rms}}^{\max}$, and B_{rms} see (Kobylkov et al. 2019; equations 2.1 and 2.3). Orientation: behavioural experiments resulted in oriented (+) or disoriented (–) birds.

Frequency band (kHz)	species	$\bar{b}^{\max}$ $(pT(\sqrt{Hz})^{-1})$	$\bar{b}$ $(pT(\sqrt{Hz})^{-1})$	$B_{\mathrm{rms}}^{\max}$ (nT)	B_{rms} (nT)	orientation	source
10 – 5000	ER	0.07	~0.02	1.18	~0.37	+	Engels et al. 2014 (Fig. 4f, blue trace)
10 – 5000	ER	5.58	~1.8	23.2	~7.35	-	Engels et al. 2014 (Fig. 4f, red trace)
20 – 450	ER	30.1	~9.5	23.1	~7.29	-	Engels et al. 2014 (Fig. 4f, green trace)
600 – 3000	ER	1.35	~0.43	2.3	~0.73	-	Engels et al. 2014 (Fig. 4f, black trace)
0.1 – 100	BC	23.3	6.6	7.98	2.26	+	Kobylkov et al. 2019 (Fig. 1a, RF-on)
0.1 – 100	BC	0.92	0.32	1.29	0.51	+	Kobylkov et al. 2019 (Fig. 1c, RF-off)
75000 – 85000	BC	1.647	0.681	5.615	2.318	-	present study (Fig. 2A, RF)
75000 – 85000	BC	0.01	0.004	0.031	0.013	+	present study (Fig. 2B, Control)

Table SI 2

Experimental result summary for the individual birds in the respective test conditions (NMF: natural magnetic field in Oldenburg; CMF: changed magnetic field, turned by 120° counter-clockwise; suffix "-RF": magnetic field condition with 75-85 MHz broadband RF fields present). For each individual (Ring) the mean orientation (*dir*), and Rayleigh value (*r*) for this orientation, the portions of valid (*valid*), random (*rnd*) and not active (*na*) trials for the overall number of trials (*N*) are listed. Only result with sufficient valid trials and a directed orientation (*valid* ≥ 3; $r \geq 0.2$) were used for the final statistics and diagrams.

	NMF						CMF						NMF-RF						CMF-RF						
Ring	dir	r	valid	rnd	na	N	dir	r	valid	rnd	na	N	dir	r	valid	rnd	na	N	dir	r	valid	rnd	na	N	Year
11 white/grey	21.09	0.42	6	1	1	8	353.11	0.39	5	0	0	5	73.21	0.83	5	2	1	8	351.70	0.51	3	0	2	5	2019
15 blue	30.57	0.91	3	2	1	6	235.76	0.29	5	0	0	5	80.00	0.76	3	0	3	6	296.89	0.28	3	0	3	6	2019
17 grey	123.79	0.36	3	1	1	5	84.03	0.25	3	0	3	6	70.00	1.00	1	2	2	5	180.00	0.64	2	1	3	6	2019
19 blue	304.87	0.20	3	1	2	6	219.36	0.30	3	1	2	6	187.63	0.73	3	2	1	6	138.95	0.84	3	0	3	6	2019
19 grey	12.93	0.80	3	1	1	5	311.33	0.65	5	0	0	5	224.76	0.27	4	0	2	6	230.00	1.00	1	1	5	7	2019
22 grey	90.00	0.91	3	1	4	8	258.44	0.41	3	2	1	6	271.44	0.80	3	1	4	8	86.78	0.48	3	0	3	6	2019
27 blue	203.91	0.15	4	3	0	7	301.79	0.27	4	1	0	5	73.59	0.40	7	0	0	7	301.97	0.22	5	0	0	5	2019
31 green	43.07	0.84	3	4	1	8	10.16	0.95	5	0	0	5	275.00	0.77	2	2	4	8	138.47	0.38	4	0	2	6	2019
32 grey	97.00	0.92	3	1	4	8	125.00	1.00	2	2	4	8	185.00	0.82	2	4	2	8	2.50	0.53	3	2	3	8	2019
33 grey	19.56	0.28	3	1	1	5	34.88	0.32	3	1	2	6	320.00	0.33	3	0	2	5	178.92	0.44	4	0	2	6	2019
34 orange/black	208.55	0.70	4	2	1	7	255.51	0.86	3	1	1	5	134.02	0.49	4	3	0	7	135.00	1.00	1	2	3	6	2019
40 grey/white	82.16	0.37	4	1	1	6	182.90	0.17	4	1	3	8	125.00	0.09	2	1	4	7	219.28	0.68	5	0	3	8	2019
45 blue	285.00	0.98	2	7	1	10	335.81	0.44	5	0	0	5	324.39	0.47	8	2	0	10	217.54	0.31	5	1	0	6	2019
47 blue	275.00	0.71	2	1	1	4	152.50	0.92	2	1	4	7	101.87	0.37	4	0	1	5	299.60	0.86	3	2	2	7	2019
49 blue	66.43	0.67	3	1	3	7	41.99	0.53	4	1	1	6	238.40	0.48	3	0	4	7	230.93	0.68	3	1	2	6	2019
58 blue	356.52	0.35	3	2	4	9	315.00	0.38	3	1	1	5	342.50	0.54	2	3	4	9	140.00	0.34	2	1	4	7	2019
64 blue	261.26	0.24	5	0	2	7	123.06	0.52	3	1	3	7	232.50	0.99	2	1	4	7	18.91	0.50	5	0	2	7	2019
67 orange	73.19	0.20	6	2	1	9	4.56	0.31	5	1	0	6	301.00	0.04	8	1	0	9	157.50	0.13	2	4	0	6	2019
79 green	174.54	0.53	4	2	3	9	319.80	0.92	3	1	2	6	30.00	0.50	2	6	1	9	137.50	0.92	2	2	3	7	2019
81 green	72.66	0.46	3	2	0	5	359.45	0.40	5	0	0	5	196.94	0.52	3	3	0	6	3.30	0.49	5	0	0	5	2019
19 violet	228.60	0.05	14	2	0	16	163.06	0.05	14	2	0	16	110.55	0.33	7	1	0	8	151.74	0.41	8	0	0	8	2021
23 yellow	164.75	0.48	9	0	1	10	253.27	0.24	11	0	1	12	307.85	0.16	7	0	1	8	335.73	0.20	12	0	0	12	2021
36 violet	81.80	0.20	10	1	3	14	311.98	0.21	12	2	0	14	260.84	0.26	7	0	1	8	172.20	0.31	8	1	1	10	2021
41 violet	164.17	0.45	7	0	3	10	205.63	0.43	6	0	3	9	168.22	0.21	5	0	3	8	217.61	0.32	8	0	6	14	2021
43 violet	152.17	0.45	8	1	1	10	298.95	0.28	6	2	0	8	98.47	0.28	9	3	2	14	215.94	0.11	15	1	2	18	2021
47 yellow	98.73	0.28	4	0	0	4	144.94	0.24	6	0	1	7	187.50	0.23	4	0	0	4	117.84	0.20	4	0	0	4	2021
55 yellow	124.30	0.39	7	1	0	8	348.88	0.30	12	0	0	12	37.39	0.37	6	1	0	7	285.11	0.23	12	0	0	12	2021
58 red	34.57	0.52	5	3	0	8	307.34	0.34	6	2	0	8	35.67	0.22	7	3	0	10	25.99	0.28	5	4	1	10	2021
59 red	58.99	0.40	5	1	0	6	241.77	0.49	5	5	0	10	279.11	0.32	5	5	0	10	28.88	0.26	5	4	1	10	2021
62 orange	200.08	0.32	9	1	0	10	19.45	0.15	12	2	0	14	293.51	0.30	7	1	0	8	117.13	0.54	8	0	0	8	2021
71 violet	158.21	0.58	5	1	0	6	345.04	0.08	12	1	2	15	190.00	0.04	4	0	0	4	66.16	0.40	7	0	1	8	2021
72 violet	95.17	0.82	3	1	6	10	325.00	0.43	3	1	4	8	141.39	0.36	3	0	3	6	240.44	0.28	3	0	7	10	2021
74 violet	347.64	0.15	13	0	1	14	205.06	0.50	9	0	1	10	281.23	0.45	8	0	0	8	153.14	0.18	13	1	2	16	2021
76 violet	337.54	0.23	4	0	0	14	345.86	0.23	5	0	8	13	94.63	0.53	5	1	4	10	71.15	0.37	5	0	5	10	2021
77 violet	305.32	0.36	8	0	2	10	208.05	0.32	9	0	4	13	65.00	0.20	5	1	4	10	53.22	0.47	5	0	5	10	2021
82 violet	78.20	0.71	5	3	2	10	335.36	0.45	7	1	0	8	154.64	0.39	6	1	3	10	154.57	0.40	7	3	0	10	2021
86 violet	356.29	0.63	5	0	3	8	213.10	0.20	8	0	6	14	303.56	0.30	6	0	4	10	22.47	0.23	7	0	1	8	2021
